# High resolution spatiotemporal patterns of seawater temperatures across the Belize Mesoamerican Barrier Reef

**DOI:** 10.1038/s41597-020-00733-6

**Published:** 2020-11-16

**Authors:** Brian Helmuth, James J. Leichter, Randi D. Rotjan, Karl D. Castillo, Clare Fieseler, Scott Jones, Francis Choi

**Affiliations:** 1 Marine Science Center, Northeastern University, Nahant, MA 01908-1557 USA; 2 Scripps Institution of Oceanography, University of California San Diego, La Jolla, CA 92093-0227 USA; 3 Department of Biology, Boston University, Boston, MA 02215-4775 USA; 4 Department of Marine Sciences, University of North Carolina at Chapel Hill, Chapel Hill, NC 27599-3300 USA; 5 Science, Technology, & International Affairs, School of Foreign Service, Georgetown University, Washington, DC 20011 USA; 6 Smithsonian Marine Station, Fort Pierce, FL 34949 USA

**Keywords:** Ecological modelling, Marine biology, Climate and Earth system modelling, Physical oceanography, Projection and prediction

## Abstract

Coral reefs are under increasingly severe threat from climate change and other anthropogenic stressors. Anomalously high seawater temperatures in particular are known to cause coral bleaching (loss of algal symbionts in the family Symbiodiniaceae), which frequently leads to coral mortality. Remote sensing of sea surface temperature (SST) has served as an invaluable tool for monitoring physical conditions that can lead to bleaching events over relatively large scales (e.g. few kms to 100 s of kms). But, it is also well known that seawater temperatures within a site can vary significantly across depths due to the combined influence of solar heating of surface waters, water column thermal stratification, and cooling from internal waves and upwelling. We deployed small autonomous benthic temperature sensors at depths ranging from 0–40 m in fore reef, back reef, and lagoonal reef habitats on the Belize Mesoamerican Barrier Reef System from 2000–2019. These data can be used to calculate depth-specific climatologies across reef depths and sites, and emphasize the dynamic and spatially-variable nature of coral reef physical environments.

## Abstract (summary)

**Table:** 

Measurement(s)	temperature of water
Technology Type(s)	Temperature Sensor Device
Factor Type(s)	temporal interval • geographic location • depth
Sample Characteristic - Environment	sea • marine reef • coral reef • marine coral reef back reef • marine coral reef fore reef
Sample Characteristic - Location	Belize Barrier Reef

Machine-accessible metadata file describing the reported data: 10.6084/m9.figshare.13095506

## Background & Summary

Reef-building corals and their algal symbionts (family Symbiodiniaceae) are highly sensitive to changes in temperature^1,2^ and in particular to deviations from local climatic norms^3,4^. Increases in seawater temperature of only one or a few degrees above a bleaching threshold can lead to the breakdown of the relationship between coral hosts and their photosynthetic algal symbionts in a process known as coral bleaching or dysbiosis, which often cascades into widespread coral mortality^5^ and subsequent reef ecosystem collapse^6^. The application of remotely sensed sea surface temperature (SST) data has served as a valuable predictor of coral bleaching, especially the metric of Degree Heating Weeks that incorporates both the magnitude and duration of excursions in temperature above regional climatological norms^3,4^.

However, susceptibility to bleaching is both patchy in space and time and is affected strongly by local factors^7,8^ such as reduced water flow that can lead both to increased localized heating^9–11^ and decreased gas exchange^12^. Further, temperatures often vary sharply with depth on coral reefs^13^ as a result of shallow-water solar heating^14^, and from the delivery of deeper, colder water from upwelling^5^ and internal waves^15–17^. What is less well understood is how temperature patterns change with depth over coral reefs^18,19^. Specifically, it is not clear if and when SST is diagnostic of temperatures at deeper locations on the same reef^20^. Furthermore, it is not well understood whether deviations from surface-recorded climatic norms necessarily reflect deviations from the depth-specific climatic norm^13,14,19^.

There is an urgent need to calibrate our understanding of the relationship between surface temperatures and benthic temperatures at specific reef locations. First, bleaching is correlated not only with the temperature, *per se* but rather with the magnitude and duration of deviations in temperature from the local climatic norm. The “bleaching threshold,” an analytical construct but one that has proved useful in bleaching predictions, is generally defined as 1 °C above the climatological mean SST of the warmest month^21^. Degree Heating Weeks is used as a cumulative measure combining both the extent and duration of heating above this bleaching threshold over the prior 12 weeks^22,23^. Defined in units of °C-time, the magnitude of the deviation from the bleaching threshold is multiplied by the duration that temperature remains above the threshold over a predetermined time window. For example, the cumulative number of degree days over any 12-week period is often chosen to indicate the likelihood of bleaching. Recent refinements of this approach have been proposed, including the inclusion of taxon-specific vulnerability to temperature anomalies^24^ and the suggestion to use *in situ* data collected at depth to calculate depth-specific climatologies and anomalies^17,19^.

Second, in the face of increasing coral mortality from thermal stress and other climate-related stressors, increasing attention has been paid to the concept of “rescue sites”^25^. By providing thermal refugia during heat waves^26^, these rescue sites may serve as larval sources that permit the repopulation of nearby assemblages that have experienced localized mass mortality^25,27^ (but see^28^). In addition to adjacent or nearby rescue sites, it has been proposed that deeper reefs (down to the mesophotic zone) may serve as reservoirs capable of rescuing their shallow counterparts with larval re-supply^29^. Whether or not deeper reefs could actually serve as rescue sites, however, depends heavily on whether the magnitude of temperature anomalies are lower at depth than they are at the surface^19^, as well as whether corals adapted to cooler temperatures at depth could effectively repopulate shallower and warmer locations. Our ability to detect potential rescue sites or reservoirs would be much better informed with an accurate understanding of whether deviations at different depths occur in synchrony with thermal stresses at or near the sea surface.

Here, we present 15 years of seawater temperature data collected *in situ* using small autonomous temperature loggers over a range of depths (0–40 m) and in fore reef, back reef, and lagoonal reef environments on the Belize Mesoamerican Barrier Reef System. This long-term dataset provides a novel mechanism for exploring the climatology on a site- and depth-specific basis.

## Methods

### Logger deployment sites

**Fig. 1:**
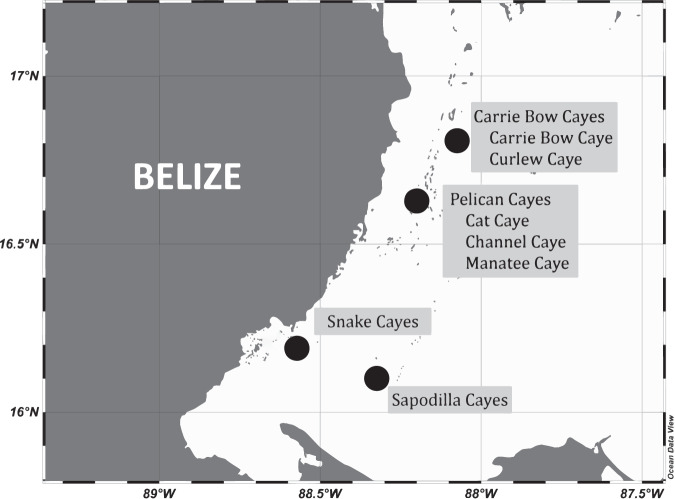
Map of logger deployment sites in Belize.

Temperature data loggers were deployed across multiple depths at 2 different locations (Carrie Bow Caye and Curlew Caye) near the Smithsonian Institution’s Carrie Bow Caye field station, (16° 48′ 08″ N, 88° 04′ 49″ W); at 3 different locations (Cat, Channel and Manatee Cayes) near the Pelican Cayes, (16° 38′ 59″ N, 88° 11′ 54″ W); and at one location each near the Sapodilla Cayes (16° 07′ 06″ N, 88° 15′ 26″ W), and Snakes Cayes (16° 11′ 30″ N, 88° 34′ 20″ W). These four different groupings of Cayes (Islands) extend from the central to southern portions of the Belize Mesoamerican Barrier Reef System (MBRS) (Fig. 1). The benthic community of the fore reef near Carrie Bow Caye represents quintessential Belizean Barrier Reef structure. It hosts four geographically distinct reef zones in close proximity: a low-relief spur-and-groove zone, the inner reef slope, the outer ridge, and the forereef slope^30^. Visiting researchers to the Smithsonian research station have documented 41 species of Scleractinian corals and 51 sponge species^31^. There are different abundances and distributions of sponge and coral species across each zone, which are thought to be driven by environmental factors^30,31^.

**Fig. 2:**
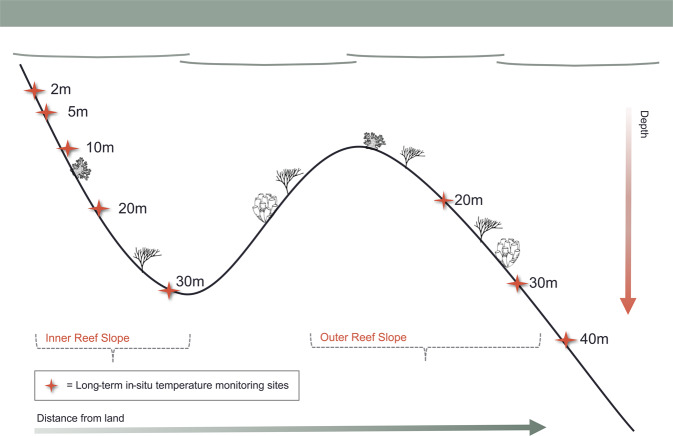
Cross-sectional view of Carrie Bow Caye describing back reef and the two fore reefs in this area: inner fore reef and outer fore reef.

These sites represent a diversity of environmental conditions including exposed fore reefs with predicted upwelling (barrier reef), buffered and sheltered sites (lagoonal environments), and areas of comparatively high flow^30^. Near Carrie Bow Caye, loggers were deployed on a double shelf on the fore reef (Fig. 2). On the inner shelf (more proximal to the reef crest) loggers were installed at five depths ranging from 2 to 28 m (~6 to 90 ft), and within the outer shelf, (towards the Cayman Trench) loggers were installed at three additional depths ranging from 18 to 37 m (~60 to 120 ft; Fig. 2). Additional loggers were deployed at Curlew Caye, approximately 1.6 km south of the Carrie Bow Caye field station, at four depths ranging from 5 to 20 m (~15–60 ft). At the Pelican Cayes, loggers were installed on the inside and the immediate outside portions of mangrove lagoons at Cat, Channel, and Manatee Cayes (Online-only Table 1). In the Sapodilla Cayes, loggers were deployed on the fore reef at White Reef at 3 m (~10 ft depth). At the Snake Cayes, loggers were deployed at 3 m (~10 ft depth) near Lagoon Snake Caye. Note that for simplification, while loggers were deployed at fixed intervals using non-metric depths we hereafter refer to depths using the approximate metric values (e.g. 10 m instead of 30 ft). All depths are referenced to the depth of the seafloor

### *In situ* seawater temperature measurements

A series of small, autonomous data loggers (StowAway, Tidbit, and HOBO Water Temp Pro loggers) manufactured by the Onset Computer Corporation (Pocasset, MA) with an accuracy of ± 0.2 °C and resolution 0.02 °C and 5 min response time were used to record temperature at all the study sites. Each instrument made seawater temperature measurements every 0.5 seconds and recorded 10 min averages (except for Hobo Water Temperature Pro loggers which acquired instantaneous measurements at 10 or 15 min intervals) from 2000–2019. At all sites deeper than 2 m loggers were affixed to a bottom-mounted float that suspended the logger 1 m above the seafloor, but the depths recorded for these loggers are referenced to the seafloor. At shallower sites (0 and 2 m) loggers were affixed to coral rubble or mangrove roots using cable ties, with the exception of the 2 m and surface loggers in the protected backreef of Carrie bow Caye, which we affixed to floats. Delayed start times and deployment of multiple loggers per site permitted deployment times of up to a year, at which time loggers were replaced. Overlapping records by multiple loggers at each location also permitted cross-calibration. All loggers were certified for accuracy by the manufacturer (Onset Computer Corporation) and were tested for accuracy following each retrieval (See *Technical Validation* below).

## Data Records

Data from all loggers are archived in two databases. The first is an interactive database maintained by Northeastern University: http://www.northeastern.edu/helmuthlab/belize/database.html. This database provides a map-based, user-friendly interface for unrestricted access to data. Data can be filtered, viewed, and downloaded by selecting a series of map layers: depth, site location, and reef type on the ESRI web-based map. Given the range of selections, the database provides the range of years over which data meeting those criteria are available for download. Metadata for each dataset are included as a downloadable spreadsheet, which includes, for each site: Country (BZ), District (a subnational jurisdiction in Belize), Site Name, and GPS coordinates (see the associated Metadata Record). The metadata file also includes information specific to each microsite, including reef location, depth of each logger, and deployment years. This database also provides an http option for SQL access. Data from each query can be downloaded as raw .csv data.

Raw data in text file format are archived in a second database hosted by Figshare Digital repository^32^. The database is organized through a series of subfolders that follows the same query order as the interactive database hosted by Northeastern University. An associated metadata spreadsheet is also included as a downloadable zip file. Each datum is named with information that is specific to the logger information: a 6-10 letter/number naming convention as follows: 2 letter country code, two-letter regional code, 2 letter site code, and numerical digits to identify microsite location (Table 1). While all data are from Belize (BZ), the inclusion of country code is to ensure interoperability with a related database^33^.

**Table 1:** Naming convention for each site.

Country code	District code	Site code
BZ = Belize	SC = Stann Creek	CB = Carrie Bow Caye
		CL = Curlew Caye
		CC = Cat Caye
		CH = Channel Caye
		MC = Manatee Caye
	TD = Toledo	SC = Sapodilla Cayes
		SN = Snake Cayes

## Technical Validation

All loggers were factory calibrated before their initial deployment. For each deployment, multiple loggers at each depth/site recorded for overlapping intervals of up to a week. Differences of more than 0.2 °C between the two loggers were flagged as potential drift and the logger was tested further in the laboratory. In rare instances where drift was detected (greater than 0.5 °C difference between adjacent loggers), logger data were discarded. On average, + /−0.05 °C differences were found across loggers deployed simultaneously at the same location. This is within the accuracy range (0.2 °C) of the manufacturer.

Data retrieved from the field were cropped to remove any data recorded by the loggers during deployment and retrieval. This was done by matching the recorded deployment and retrieval dates and times with the timestamps of the loggers. In addition, quality control for each time series was accomplished by flagging unrealistic temperature drops or rises in the dataset, which we defined as a change of greater than 1 °C in a single (10–15 min) time step. These errors can occur when loggers undergo physical trauma. Flagged data were removed from the dataset.

## Usage Notes

The majority of the Belizean Barrier Reef is experiencing “a phase of transition” toward a community dominated by macroalgae and coral rubble^31^, similar to reefs throughout the Caribbean. Total coral cover across all reef zones has decreased by 90% since 1995 with reef-building corals now comprising less than 8% of benthic cover^31^. Sponges remain present, covering about 3% of the benthos, a slight increase since the 1990s. The dramatic decline of coral species near Carrie Bow Caye likely has multiple drivers. McField^34^ first established bleaching-induced tissue mortality as a driver across the wider barrier reef. Villamizar *et al*.^31^ proposed hurricane activity as a driver decreasing physical structure and increasing rubble abundance in the benthic community near Carrie Bow Caye, noting eight hurricanes that have struck Belize with force since 1993. However, the relative importance of *in situ* temperature change at multiple depths as a driver of this transition has not been fully established.

This high temporal resolution dataset is valuable for examining sub-daily variability (daily ranges recorded by some loggers was as high as 3–4 °C) at different depths that traditional SST datasets cannot provide. The resolution of 10–15 mins is also valuable as a means of observing discrete events, including internal waves that are rarely detectable in hourly datasets and are only detectable from the surface under limited conditions^35^.

**Fig. 3:**
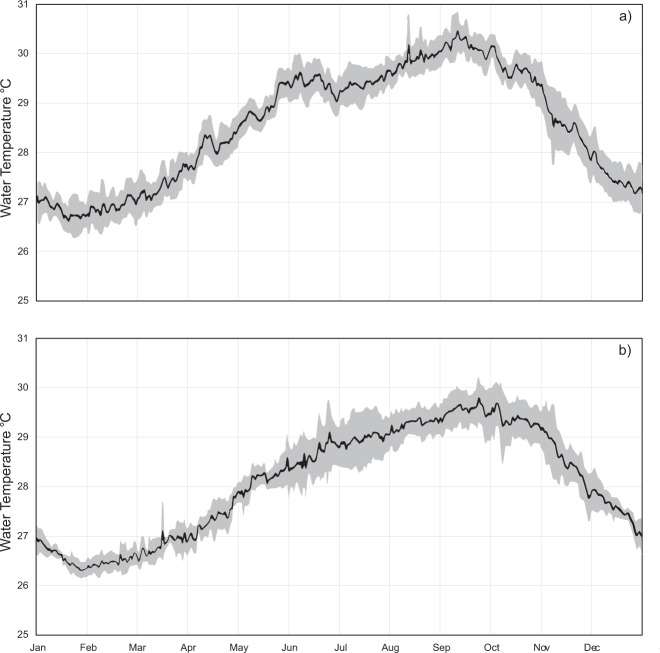
Climatology of daily maximum water temperature recorded at (**a**) 5 m depth inner reef slope and (**b**) 40 m depth outer reef slope at Carrie Bow Caye from 2001–2006, calculated as the average of each day of the year. Shaded areas represent 95% confidence intervals of interannual temperature variation of each day of the year.

Furthermore, deeper (e.g. depth >15 m) temperature series on coral reefs are relatively rare, generally of short duration, and/or often interrupted by data gaps associated with funding constraints, shifting research priorities, and instrument losses and failures. While our data are also subject to some of these issues, they nevertheless provide relevant analysis opportunities due to their longevity and large distribution of sites and depths. One example of an application of this data set is to produce depth-specific temperature climatologies that can be used to predict depth-specific bleaching threshold temperatures (Fig. 3) and can be combined with time series of biological observations to predict and test taxon-specific bleaching patterns in relation to temperature stresses. Long-term data sets also provide a means of assessing the spatiotemporal frequencies over which measurements conducted at one location and depth are correlated with or decoupled from patterns at other locations. For example, analyses of the extent of frequency-specific correlation, or coherence, of temperature variation among depths can be conducted. Combining these records with remotely sensed SST observations can also provide insight into the ability to predict or approximate thermal extremes at depth from remotely sensed observations.

## Online-only Table

**Online-only Table 1:** Metadata information of logger deployment.

Country	Cayes	Site	Site name	Habitat	Depth (m)	Latitude	Longitude	Deployment Period
Belize	Carrie Bow Cayes	Carrie Bow Caye	BZSCCB11	Back reef	0	16.80127	−88.082072	2000–2005
Belize	Carrie Bow Cayes	Carrie Bow Caye	BZSCCB8	Back reef	2	16.80127	−88.082072	2000−2015, 2017−2019
Belize	Carrie Bow Cayes	Carrie Bow Caye	BZSCCB9	Fore reef inner	2	16.8009	−88.079817	2000−2012
Belize	Carrie Bow Cayes	Carrie Bow Caye	BZSCCB3	Fore reef inner	5	16.8009	−88.079817	2001−2009, 2011−2012, 2015−2018
Belize	Carrie Bow Cayes	Carrie Bow Caye	BZSCCB4	Fore reef inner	10	16.79972	−88.07863	2000−2014, 2017−2018
Belize	Carrie Bow Cayes	Carrie Bow Caye	BZSCCB6	Fore reef inner	20	16.79898	−88.0776	2000−2014, 2017−2018
Belize	Carrie Bow Cayes	Carrie Bow Caye	BZSCCB5	Fore reef inner	30	16.79898	−88.0776	2000−2004
Belize	Carrie Bow Cayes	Carrie Bow Caye	BZSCCB10	Fore reef outer	20	16.79837	−88.07683	2000−2011, 2017
Belize	Carrie Bow Cayes	Carrie Bow Caye	BZSCCB2	Fore reef outer	30	16.79837	−88.07683	2000−2006, 2017−2019
Belize	Carrie Bow Cayes	Carrie Bow Caye	BZSCCB7	Fore reef outer	40	16.79837	−88.07683	2000−2006, 2017
Belize	Carrie Bow Cayes	Curlew Caye	BZSCCL1	Fore reef	5	16.77592	−88.07653	2017
Belize	Carrie Bow Cayes	Curlew Caye	BZSCCL2	Fore reef	10	16.77592	−88.07653	2017−2019
Belize	Carrie Bow Cayes	Curlew Caye	BZSCCL3	Fore reef	15	16.77632	−88.0748	2017−2019
Belize	Carrie Bow Cayes	Curlew Caye	BZSCCL4	Fore reef	20	16.77632	−88.0748	2017−2019
Belize	Pelican Cayes	Cat Caye	BZSCCC5	Lagoon	0	16.652092	−88.199404	2000−2001
Belize	Pelican Cayes	Cat Caye	BZSCCC1	Lagoon	2	16.652092	−88.199404	2001−2002
Belize	Pelican Cayes	Cat Caye	BZSCCC6	Back reef	0	16.651963	−88.196906	2000−2002
Belize	Pelican Cayes	Cat Caye	BZSCCC4	Back reef	2	16.651963	−88.196906	2001−2002
Belize	Pelican Cayes	Cat Caye	BZSCCC2	Back reef	5	16.651963	−88.196906	2001−2003, 2004−2005
Belize	Pelican Cayes	Cat Caye	BZSCCC3	Back reef	10	16.651963	−88.196906	2001−2003, 2004−2005
Belize	Pelican Cayes	Channel Caye	BZSCCH1	Back reef	5	16.662761	−88.13632	2001−2002, 2004−2005
Belize	Pelican Cayes	Channel Caye	BZSCCH2	Back reef	10	16.662761	−88.13632	2001−2002, 2004−2005
Belize	Pelican Cayes	Manatee Caye	BZSCMC3	Lagoon	5	16.666483	−88.192316	2000−2002
Belize	Pelican Cayes	Manatee Caye	BZSCMC1	Back reef	5	16.665048	−88.187625	2000−2002
Belize	Pelican Cayes	Manatee Caye	BZSCMC2	Back reef	10	16.665048	−88.187625	2000−2002
Belize	Snake Cayes	Snake Caye	BZTDSN1	Back reef	5	16.19172	−88.57228	2002−2007
Belize	Sapodilla Cayes	Sapodilla Caye	BZTDSC1	Fore reef	5	16.108138	−88.26666	2002−2007
